# Spectrophotometric Determination of the Ionization Constant of 2,4,6-Trinitro-*m*-cresol in Water at 25 °C

**DOI:** 10.6028/jres.064A.055

**Published:** 1960-12-01

**Authors:** Marion Maclean Davis, Maya Paabo

## Abstract

The ionization constant of 2,4,6-trinitro-*m*-cresol in water at 25 °C was determined by a spectrophotometric procedure. The *p*K value 0.81 (K ≈ 0.16) was obtained.

## 1. Introduction

J. Kendall [1]^1^ determined the conductivity of 2,4,6-trinitro-*m*-cresol (methylpicric acid) and its sodium salt in water, and concluded that a saturated solution (0.01–*M*) at 25 °C is about 92-percent dissociated. Applying the method of calculation described in [2] to these data gives a *p*K value of about 1 for trinitro-*m*-cresol. Values of *p*K for trinitro-*m*-cresol can be estimated by assuming that effects of substituents on the ionization constants of phenols are additive [2]. For example, by applying data for phenol and its monosubstituted derivatives the estimated *p*K value 1.66 is obtained. Data for 2,4-dinitropbenol, *o*-nitrophenol, and *m*-cresol lead to the *p*K value 1.41. A lower *p*K value, 0.95, is obtained by using *p*K values for 2,6-dinitrophenol, *p*-nitrophenol, and *m*-cresol. However, a substantially higher *p*K value (2.8) was obtained by a potentiometric procedure [3].

This paper describes the determination of *p*K trinitro-*m*-cresol by a spectrophotometric procedure.

## 2. Method and Results

Commercial 2,4,6-trinitro-*m*-cresol of high purity was recrystallized successively from water, aqueous ethanol, and benzene-cyclohexane, and then dried at 80 °C for 2 hr; mp, 107.0 to 107.5 °C. Potentiometric weight-titrations indicated the purity was greater than 99.8 percent.

**Figure 1:**
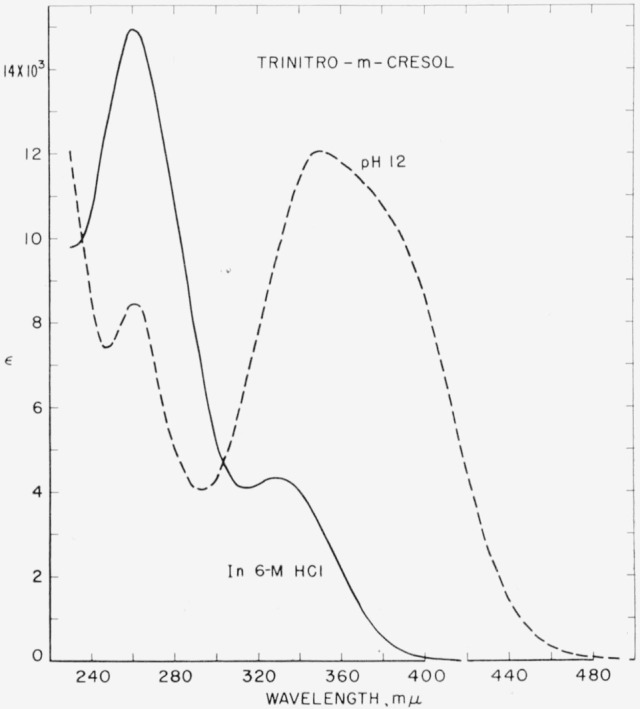
Spectral absorption curves of 2,4,6-trinitro-*m*-cresol in aqueous acid (approx. 6–*M HCl*) and in aqueous alkali (*NaOH*, *pH* ≈12). The curve for a solution containing 4.5–*M* HCl was almost identical with that shown for 6–*M* hydrochloric acid solution.

The spectrophotometric procedure used for determining *p*K was closely analogous to that described in [2]. In figure 1 molar absorption curves for ionized and nonionized trinitro-*m*-cresol are shown. Additional absorption curves were obtained in which the hydrochloric acid content varied from about 0.05–M to 6*–M.* The two isosbestic points shown in figure 1 remained fixed throughout.

Values of *p*K were calculated from absorbance measurements at 350 m*μ*, 380 m*μ*, and 410 m*μ*. The data and the calculated values of *p*K are summarized in table 1. These yield the average *p*K value 0.806, or approximately 0.81. The corresponding ionization constant is 0.156.

## Figures and Tables

**Table 1:** Ionization constant of 2,4,6-trinitro-*m*-cresol in water at 25 °C^a^,^b^

Concentration of HCl^b^	*D*	log[(D−*D*1)/(*D*_2_−*D*)]	−log [*H*^+^]^b^	−2 log *γ*_±_	*p*K^c^

λ = 350 m*μ* *D*_1_ =0.156, *D*_2_=0.604					

0.0483	0. 523	0.656	1.316	0.160	0. 820
.0676	.503	.536	1.170	.177	.811
.0966	.477	.403	1.015	.196	.808
.171	.424	.173	0.766	.225	.818
.214	.405	.097	.669	.234	.806
.257	.380	.000	.590	.240	.830
.343	.357	−.089	.465	.244	.798
.483	.315	−.259	.316	.242	.817

λ = 380 m*μ* *D*_1_=0.027, *D*_2_=0.538					

0.0483	0.447	0.664	1.316	0.160	0.812
.0676	.423	.537	1.170	.177	.810
.0966	.395	.410	1.015	.196	.801
.171	.337	.188	0.766	.225	.803
.214	.312	.101	.669	.234	.802
.257	.286	.012	.590	.240	.818
.343	.259	−.080	.465	.244	.789
.483	.212	−.246	.316	.242	.804

λ = 410 m*μ* *D*_1_ =0, *D*_2_=0.329					

0.0483	0.270	0.661	1.316	0.160	0.815
.0676	.257	.553	1.170	.177	.794
.0966	.237	.411	1.015	.196	.800
.171	.199	.185	0.766	.225	.806
.214	.184	.104	.669	.234	.799
.257	.165	.002	.590	.240	.828
.343	.155	−.050	.465	.244	d.759
.483	.120	−.241	.316	.242	.799

Average *p*K					0.808
K					.156

a The concentration of trinitro-*m*-cresol was 5.001×10^−5^ *M.* The optical absorption cells were 1 cm in length.

b All concentrations are in moles per liter.

c *p*K = −log[*H*+]−2 log *γ_±_−*log[(*D*−*D*_1_)/(*D*_2_−*D*)]. See [2].

d Discarded.

## Footnotes

1 Figures in brackets indicate the literature references at the end of this paper.
